# Myoepithelial Tumor of Soft Tissue With the Novel IRF2BP2::CDX1 Gene Fusion

**DOI:** 10.7759/cureus.90435

**Published:** 2025-08-18

**Authors:** Rana Naous, Ricardo Diaz-aragon, Arivarasan Karunamurthy

**Affiliations:** 1 Pathology, University of Pittsburgh Medical Center, Pittsburgh, USA; 2 Laboratory Medicine, University of Pittsburgh Medical Center, Pittsburgh, USA

**Keywords:** cdx1, irf2bp2, myoepithelial, soft tissue, vascular

## Abstract

Myoepithelial tumors of soft tissue are mesenchymal tumors with characteristic gene fusions involving the* EWSR1* or *FUS* gene in many cases. *EWSR1-*negative* *gene fusions are rarely reported in myoepithelial tumors of soft tissue. Herein, we report a novel gene fusion, *IRF2BP2::CDX1,* in a myoepithelial tumor of soft tissue in a 36-year-old female harboring rare morphologic findings, including multinucleated giant cells and microhemorrhages. Our case adds to the expanding pool of* EWSR1*-negative myoepithelial tumors of soft tissue. These rare tumors appear to share some histological features, including fascicular spindle cell arrangement with vascular changes. Our report further corroborates the clinicopathological characteristics and molecular features of such tumors.

## Introduction

Myoepithelial tumor of soft tissue is a rare mesenchymal tumor with characteristic gene fusion involving the *EWSR1* or *FUS* gene in about 50% of cases [1]. Vascular changes, multinucleated giant cells, and microhemorrhages are features not commonly described in myoepithelial tumors of soft tissue. *IRF2BP2* (interferon regulatory factor 2 binding protein 2) is a gene commonly involved in apoptosis, survival, cell differentiation, cancer development, and immune response [2]. The* CDX1 *(caudal-type homeobox 1) gene has a role in regulating enterocyte differentiation, expression of the intestinal alkaline phosphatase gene, and inhibiting beta-catenin/T-cell factor transcriptional activity [3]. *IRF2BP2* and *CDX1* gene fusions are rarely reported in myoepithelial tumors of soft tissue. Herein, we report a novel gene fusion *IRF2BP2::CDX1 *in a myoepithelial tumor of soft tissue with rare morphologic findings.

## Case presentation

**Figure 1:**
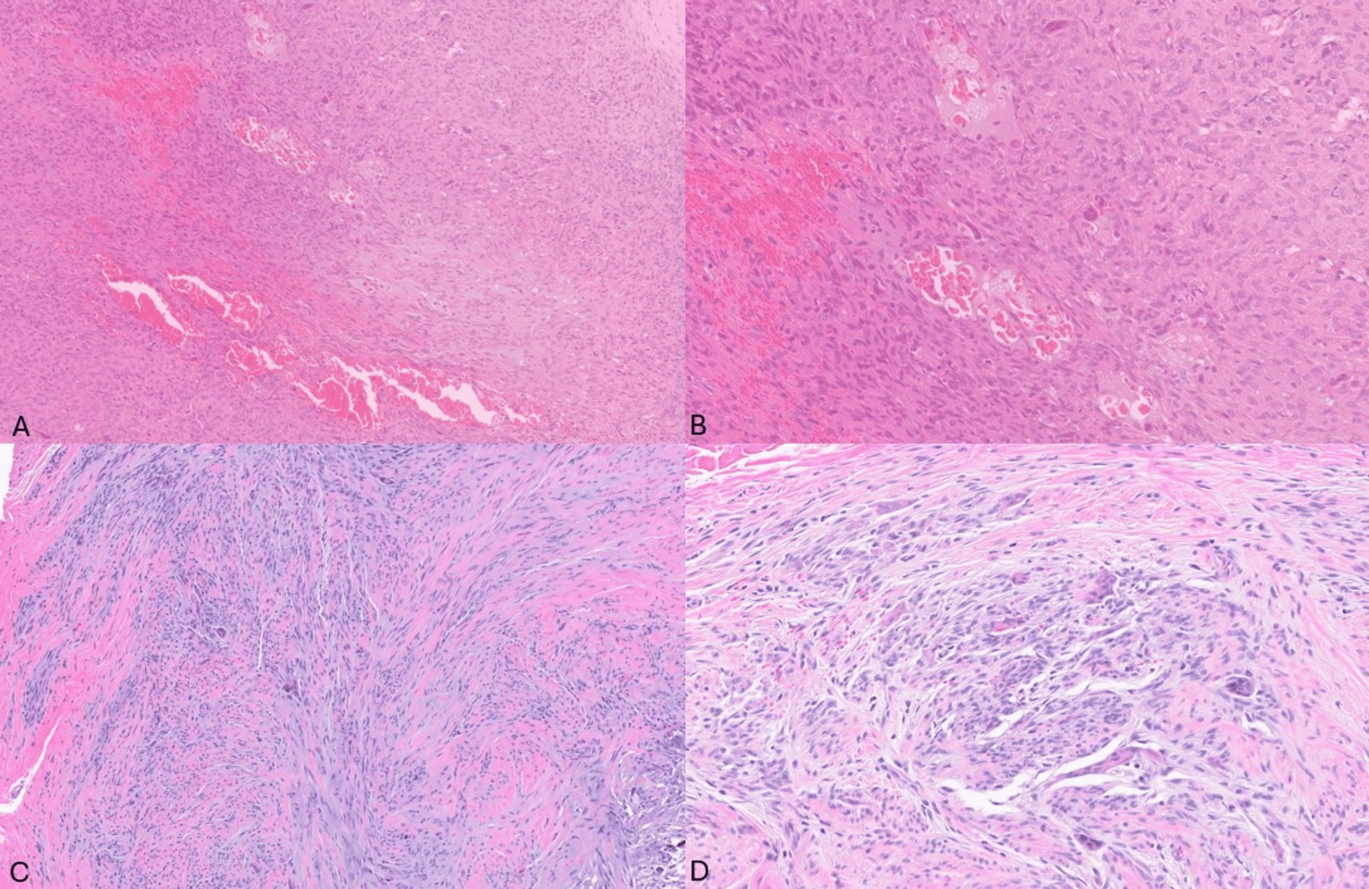
Morphologic features of the original and recurrent tumor. A-B: Original tumor demonstrating spindle cell proliferation in short fascicles set in a collagenous to myxoid stroma with microhemorrhages admixed with osteoclast-like giant cells (H&E; 10x and 20x). B-C: Recurrent tumor demonstrating similar spindle cell morphology to original tumor with osteoclast-like giant cells and extravasated red blood cells (H&E; 10x and 20x).

Our case involves a 36-year-old female with no other medical history who had a previous, around 14 years ago, marginally resected 3.5 cm myoepithelial tumor of soft tissue involving the dorsum of her right foot with a final positive margin, and currently presenting with a small lump in the middle of her prior incision compatible with recurrence. Radiologic CT imaging (images not available) demonstrated a small enhancing round mass in the web space between the second and third metatarsals. The recurrent mass was marginally excised, and histologic sections revealed a 0.3 cm mass with similar morphology to the patient’s initial tumor, characterized by a lobulated spindle cell proliferation arranged in a storiform pattern or short fascicles, admixed with osteoclast-like giant cells and set in a collagenous to occasionally myxoid stroma with occasional microhemorrhages (Figure 1A-1D).

**Figure 2:**
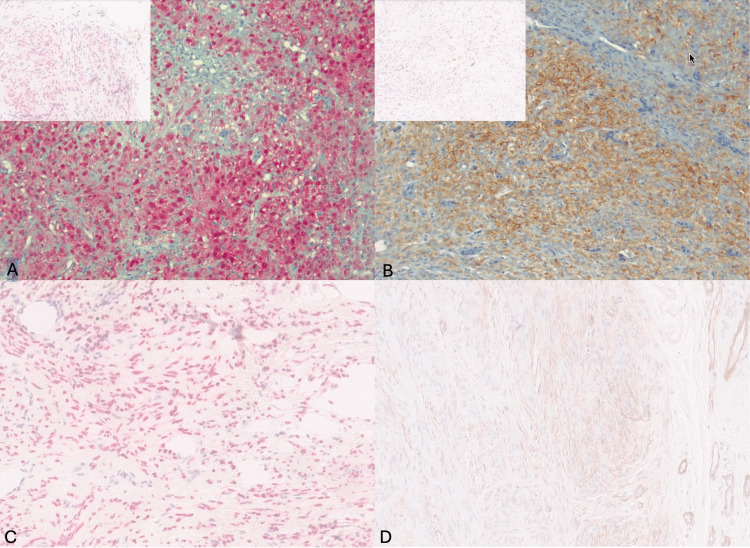
S100, cytokeratin, SOX10, and SMA immunostains in the original and recurrent tumor. A: Cytoplasmic and nuclear positivity for  S100 immunostain in the original tumor (20x) and recurrent tumor (inset). B: Cytoplasmic and membranous staining for pankeratin in the original tumor (20x) and for CK AE1/AE3 in the recurrent tumor (inset). C: Strong nuclear SOX10 positivity in recurrent tumor (20x). D: Cytoplasmic SMA staining in the recurrent tumor (20x).

The lesional cells were positive for SOX-10 and patchy positive for S-100, SMA, and cytokeratin AE1/AE3 (Figure 2A-2D).

Table 1 details the immunostain markers' extent and intensity in the original and recurrent tumor.

**Table 1:** Immunostain markers' extent and intensity in the original and recurrent tumor.

Immunostain	Original tumor (extent, intensity)	Recurrent tumor (extent, intensity)
S100	Patchy, moderate to strong	Patchy, moderate
Sox10	Not performed	Patchy, strong
Pankeratin	Patchy, moderate to strong	Not performed
CK AE1/AE3	Not performed	Patchy, weak to moderate
SMA	Patchy, weak	Patchy, weak

**Figure 3:**
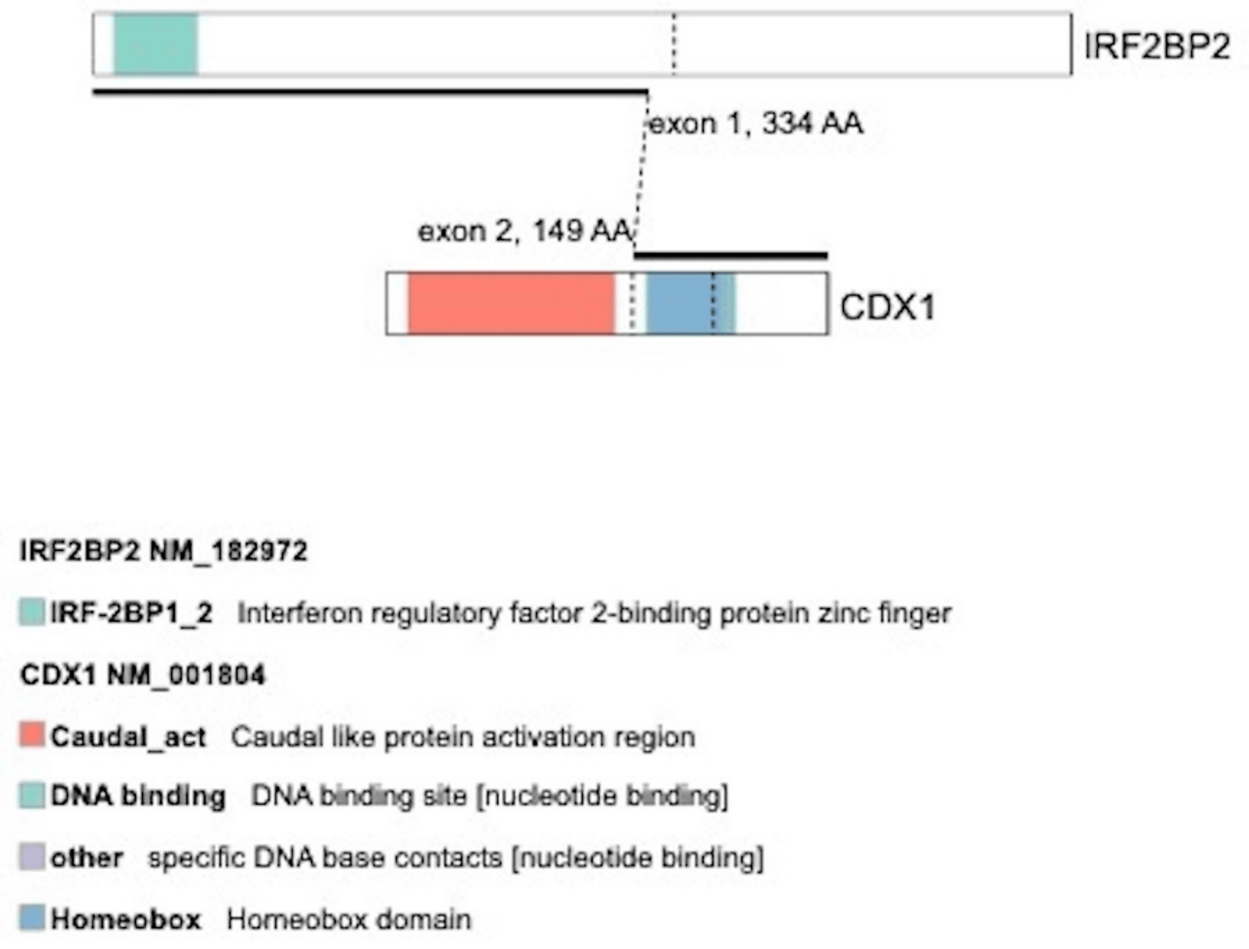
IRF2BP2::CDX1 gene fusion Graphical representation of IRF2BP2::CDX1 gene fusion involving exon 1 of the IRF2BP2 gene on chromosome 1 (5' transcript NM_001077397) and exon 2 of the CDX1 gene on chromosome 5 (3' transcript NM_001804).

The neoplasm on both the original and the recurrence had a low-grade morphology, with little to no mitotic activity, and no evidence of necrosis or significant cytologic atypia. Oncomine Comprehensive Assay v3, an amplification-based Next Generation Sequencing (NGS) assay panel (Thermo Fisher Scientific Inc., USA) targeting 161 cancer driver genes for SNV (single nucleotide variants), gene mutations, copy number alterations, and fusions, was performed on the recurrent tumor and was negative. Subsequently, whole-transcriptome RNA sequencing (RNA-Seq) was performed on the recurrent tumor to assess for any pathogenic gene fusions via Illumina Next Seq using three fusion callers (ChimeraScan, FusionCatcher, and STAR-Fusion) with a maximum read length of 2 x 150 bp and maximum reads per run of up to 1.2 billion. RNA-Seq identified a novel in-frame gene fusion (Figure 3) *IRF2BP2::CDX1,* resulting from a t(1;5)(q42;q32) translocation, involving exon 1 of the *IRF2BP2* gene and exon 2 of the *CDX1 *gene. The patient is followed up clinically with no additional recurrences thus far (2.5 months post-surgical operation). 

## Discussion

*EWSR1* is the most commonly rearranged gene in myoepithelial tumors of soft tissue, followed by *FUS,* with fusion partners including *ATF1,* *KLF15*, *KLF17*, *PBX1*, *PBX3*, *POU5F1*, *VGLL1, *and *ZNF444* [1]. *IRF2BP2* gene fusion is a unique and novel finding in myoepithelial tumors of soft tissue. *IRF2BP2::CDX1* gene fusion is predicted to code for a chimeric transcription factor containing the N-terminal zinc finger domain of *IRF2BP2* and homeobox domain of *CDX1* [4,5]. Although* the IRF2BP2::CDX1* gene fusion has been mentioned in one case of myoepithelial tumor of soft tissue [6], a complete characterization and description of such a case was not provided by the corresponding authors. A novel *IRF2BP2 *gene fusion has been described in one case of digital intravascular myoepithelioma of soft tissue, whereby *the CDX2 *gene, rather than the *CDX1* gene, was the fusion partner [4]. Our case technically would be the third myoepithelial tumor of soft tissue to harbor an IRF2BP2 gene fusion and the first fully characterized case with a novel *CDX1* gene fusion partner and rare morphologic features. 

Myoepithelial tumors of soft tissue usually present as variably sized painless masses on limbs, limb girdles, trunk, head and neck, bone, visceral organs, or skin of young to middle-aged adults with a relatively similar distribution between males and females. They grossly appear well circumscribed and multinodular with infiltrative margins microscopically. The tumor cells can be spindled, ovoid, plasmacytoid, or rhabdoid with minimal atypia and variable mitotic activity set in variably myxoid, hyalinized, or chondroid stroma. Myoepithelial carcinomas usually have moderate to severe atypia or pleomorphism with prominent nucleoli, high mitotic count, and necrosis. The tumor cells in myoepithelial tumors of soft tissue are usually positive for keratin AE1/AE3, EMA, S100, calponin, and variably positive for GFAP, smooth muscle actin, SOX10, and p63 [7,8]. Vascular changes, including microhemorrhages or papillary endothelial hyperplasia and multinucleated giant cells, are rare features not commonly reported in myoepithelial tumors of soft tissue.

The presence of multinucleated giant cells and microhemorrhages in our case was an unusual finding that is not commonly described in myoepithelial tumors of soft tissue. Vascular changes were a unique finding in the digital intravascular myoepithelioma of soft tissue case with the novel *IRF2BP2::CDX2* gene fusion [4], whereby the authors describe a similar spindle cell proliferation arranged in tight fascicles colliding with unusual areas of papillary endothelial hyperplasia in association with an organizing thrombus. Interestingly, one of the reported roles of *IRF2BP2* is as an ischemia-induced co-activator of VEGFA expression that may contribute to re-vascularization of ischemic cardiac and skeletal muscles [9], while induction of *the CDX1 *gene has been reported to cause altered transcript expression of genes related to cell adhesion and angiogenesis [3]. It is uncertain at this point in time if the presence of an *IRF2BP2* and *CDX1* gene fusion contributes to or affects the vascular milieu of myoepithelial tumors of soft tissue. More cases are needed to further elucidate such a phenomenon. 

The differential diagnoses in our case, based on the tumor morphology and immunophenotype, included nodular fasciitis, fibroma of the tendon sheath, and spindle cell melanoma. The storiform and short fascicular arrangement with admixed osteoclast-like giant cells in our case mimicked nodular fasciitis and fibroma of the tendon sheath; however, the absence of USP6 gene rearrangement and the dual immunopositivity for S100 and SOX10 argued against both entities, but rather raised consideration for spindle cell melanoma. However, the absence of melanoma markers, including HMB45 and Melan-A, and the negative molecular profile for any melanoma-associated genetic alterations, including BRAF, NRAS, and NF1 gene alterations, argued against such an entity. 

Per the WHO, myoepithelial tumor of soft tissue is a rarely metastasizing tumor of borderline biologic potential with a 20% local recurrence risk, especially if incompletely excised. In our case, the original tumor was marginally excised with a positive final surgical resection margin, and despite that, it recurred after 14 years, thus reflecting the tumor’s indolent behavior. As the recurrent tumor was similar morphologically to the original tumor, it is safe to assume that it will behave in a similar indolent fashion, with surgical excision being currently the main treatment of choice.

## Conclusions

Our case adds to the expanding pool of* EWSR1*-negative myoepithelial tumors of soft tissue. These rare tumors seem to share some histological features, including fascicular spindle cell arrangement with vascular changes. Our report further corroborates the clinicopathological characteristics and molecular features of such tumors. Given its rarity, the prognostic significance of the *IRF2BP2::CDX1* gene fusion in myoepithelial tumors of soft tissue is uncertain, with surgical excision being the mainstay of treatment in morphologically low-grade tumors at this point in time.
